# Making De-Extinction Mundane?

**DOI:** 10.1371/journal.pbio.1001825

**Published:** 2014-03-25

**Authors:** Carrie Friese, Claire Marris

**Affiliations:** 1 Sociology Department, London School of Economics and Political Science, London, United Kingdom; 2 Department of Social Science, Health and Medicine, King's College London, London, United Kingdom; King's College London, United Kingdom

## Abstract

Previous debates on cloning endangered animals provide useful lessons for how de-extinction could incorporate concerns from various, focusing less on spectacular science and more on daily practices.

*This Perspective is part of the Public Engagement in Science series.*

## Introduction

The use of new technologies to bring back extinct species has recently become a topic widely discussed in the media, partly as the result of a TEDx programme on de-extinction [1] at the National Geographic Headquarters, timed to coincide with a *National Geographic* cover story in April 2013. Two weeks earlier, Stuart Brand, a key proponent of de-extinction, gave his own TED talk [2]. These public events were followed by high-profile conferences at Cambridge (UK) and Stanford Universities [3],[4]. These events have begun to shape the contours of ‘de-extinction,’ by defining the relevant techniques (cloning, genome editing, back breeding, stem cell manipulation) and also the actors that can legitimately participate. Thus, de-extinction is currently crystalizing into a field that includes not only bioscientists but also, to varying degrees, the popular press, bioethicists, conservationists, and scientists from other fields (for example, synthetic biologists).

De-extinction has raised a number of ethical and political questions: Will it divert resources from other tried-and-tested measures for conservation? Will the resurrected animals be classified as members of the extinct species? Are conservationists too pessimistic and sceptical about cutting-edge science to embrace its potential? How will we ethically care for the animals used in and produced by these techniques? Are there hidden commercial interests at stake? What is striking, from our perspective, is that many of these debates have been held before: the tropes regarding de-extinction are remarkably similar to those used in debates regarding cloning endangered animals.

In this paper, we explore the relevance of previous debates and argue that important insights can be gleaned from them as de-extinction moves forward, and that there is another set of questions that has not yet been adequately addressed. In line with the arguments of Marris and Rose [5] in the opening editorial for this series “Opening Engagement: Exploring Public Participation in the Biosciences,” we examine how, in the field of cloning endangered animals, the concerns of conservationists have in some cases been the basis for reformulating scientific practices in a way that can be interpreted as a form of ‘upstream’ public engagement. We argue that de-extinction could learn valuable lessons from these earlier projects regarding how to incorporate contributions from various publics; and demonstrate what a sociological approach can add to the exploration of these questions, in ways that traditional bioethics and ELSI (Ethical, Legal and Social Implications) approaches cannot.

## Old Debates

Concerns raised about de-extinction have included: resource allocation, species identification and classification, and the relationship between technology and nature preservation. Here we discuss how these debates have been previously articulated.

Conservationists have voiced concern that de-extinction will shift financial and other forms of support from more established land management practices to biotechnological solutions [6],[7]. This was also a key concern at the turn of the twenty-first century when scientists began to successfully clone endangered and threatened animals. Indeed, shortly after the cloning of a gaur (an endangered cow species) was announced, critics of the US Endangered Species Act (ESA) began to argue that this legislation was outdated. They argued that the ESA was no longer necessary because the availability of cloning techniques meant that species would no longer go extinct, which is a discourse rooted in technological optimism that environmentalists have long been critical of [8]. The concern here is that funding will move from tried-and-tested preservation strategies to technologies that are represented as a magic bullet but that are still in the infancy stage, and are thus uncertain. For example, after 20 years of research in assisted reproduction, even ‘simple’ techniques like artificial insemination continue to be difficult to use routinely in ex situ species preservation practices [9]. This is not to say that technologies should not be developed, but that their contributions are necessarily limited in the development stage.

Questions have been raised about whether or not an extinct animal reproduced through back breeding, cloning, or genetic engineering would be classified as that species (e.g., a passenger pigeon) or a new kind of species [7],[10]. This resonates with debates on the ontological status of cloned endangered animals, which similarly rely upon the use of closely related and abundant animals as egg donors and surrogates [9],[11]. What we learn from these earlier discussions is that resurrected animals can be categorized differently by different agencies. For example, the cloned banteng—another endangered cow species that is now on display at the San Diego Zoo—is considered a hybrid by the US Fish and Wildlife Service, and is therefore not considered part of the banteng population. However, the Species Survival Plan, organized by the American Zoo and Aquarium Association in order to breed endangered species in captivity, considers this same animal a banteng and includes him in their studbook. The lesson from previous experiences with cloning endangered animals is that ontological debates within de-extinction could be better understood within the institutionalized practices through which species are managed.

De-extinction risks being framed as a two-sided debate, between technologically enthusiastic and optimistic scientists who seek to master nature versus depressed and technologically sceptical environmentalists who focus unduly on possible unintended consequences. This is a rather longstanding frame [8], and is also how the recent Cambridge meeting was portrayed [12]. A better way of pursuing discussions regarding the future of de-extinction, which takes a more sociological perspective, is to ask: What kind of nature does de-extinction seek to make? Whose interests (human and otherwise) are met through making this kind of nature? Whose interests are not met? How are resulting disparities addressed? This would allow de-extinction to be considered in context, in a manner that learns from previous engagements between conservation and technology.

## Public Forums

One of the striking features of de-extinction is that, even at this early phase, public outreach has been proactive and extensive. The far-reaching coverage of this effort in *National Geographic*, through the TEDx programme and other TEDx talks and related media coverage of these, and academic conferences attest to this. This public outreach has indeed helped to establish and define de-extinction as a scientific field and topic of public interest. Meanwhile, discussions on cloning endangered animals have, in contrast, been more conventional, largely occurring in the context of professional conferences, within zoological organizations, and in journal commentaries.

Despite the seemingly more ‘public’ nature of de-extinction, we argue that cloning endangered animals has, at times, engaged with ‘public debate’ in a manner that de-extinction could usefully learn from as it moves forward. At least some cloning experiments involving endangered animals have taken up and addressed the concerns of their critics by changing their scientific practices. For example, different kinds of cells and animals were used in different cloning experiments so that the resulting animal did not simply show that it was possible to clone, but also how cloning could be of value to species preservation efforts. After the gaur died, the San Diego Zoo decided to clone a banteng instead because he was considered more genetically valuable within contemporary ex situ preservation practices [9],[13]. In other words, the concerns of conservationists have been the basis for reformulating the experimental practice of cloning endangered animals. This raises the question: how might future de-extinction experiments be designed in order to address the concerns that have been raised over the past year? As Marris and Rose noted [5], ‘upstream’ public engagement seeks “to enable a range of actors, including lay publics, but also the widest possible range of people who might be interested or affected, to help shape the trajectory of innovation.” The TEDx programme and Stanford conference show that a range of actors have been brought together in order to discuss de-extinction. This is a laudable opening, which can now take on the challenge of bringing such diverse groups together in the conduct of de-extinction research itself.

## The Ethics of Using and Making Animals in Science

One area of sustained concern has been the ethics of using and making animals through the scientific practices associated with de-extinction. First, there are concerns about the welfare of cell donors and surrogates used to reproduce de-extinct animals. Second, there are also welfare concerns regarding the de-extinct animal itself. One example comes from cloning, where resulting animals often die in a painful manner and shortly after birth due to birth defects associated with somatic cell nuclear transfer. The cloned Spanish bucardo is often used as an example of this. This is the only animal of an extinct species to be brought back to life through de-extinction, and the animal died minutes after birth in acute respiratory distress. Third, there are more long-ranging concerns regarding where and with whom animals produced through de-extinction will live. Will the animal live in a zoo? Will it be reintroduced into a (re)wild, park-like region? The early de-extinct animals will not have any conspecifics. Who will these animal live with, whether it be in a zoo or a park? If the animal is social, what will the consequences of this be?

There are, however, another set of questions regarding making animals that arise from a social science perspective as opposed to the above bioethics and conservation perspectives. Who will take care of the newly born, de-extinct animal? Is there a group of professionals who have the knowledge required to rear the de-extinct animal? Are professionals of this group available and willing to engage in such work for de-extinction? How will they be involved in the experimentation? In this context, it is important to note that the death of the cloned gaur was raised as an example of the health problems associated with cloning at the Stanford conference. However, this animal actually died because of husbandry problems. People involved in the experiment simply did not know how to hand rear a gaur. This was another reason why the San Diego Zoo decided to clone a banteng instead of a gaur in the subsequent cloning project [9]. Zoo keepers at the park had experience hand rearing this species. A social science perspective on work and employment is able to translate some of the more abstract ethical concerns regarding the lives of animals produced through science into more tangible, organizational questions. Questions regarding animal care need to be understood as a crucial part of de-extinction experimentation, rather than downstream concerns.

## Political Economies of De-Extinction

A wider range of actors are involved in de-extinction for a variety of reasons. Those with commercial interests are at times viewed with scepticism, which can have ramifications for de-extinction more generally. For example, Robert Lanza of Advanced Cell Technology (ACT) was a key figure in cloning both the gaur and the banteng and is now pursuing de-extinction. ACT had commercial interests in cloning endangered animals; it was a means for the company to prove the principle of interspecies nuclear transfer as part of its human embryonic stem cell research programme while also gaining positive public relations because reproducing endangered animals is often considered an indisputably positive thing to do [9]. One question raised at the Stanford conference was why de-extinction is being pursued. While altruistic purposes may be highlighted, there was the clear assumption on the part of some participants that there was a commercial component to this research venture. Jake Sherkow, an expert on patent law and bioscience regulation, noted that ACT had patented the use of interspecies nuclear transfer to clone extinct animals in 2001. There is a widespread belief that commercial interests and altruism are mutually exclusive, a belief that we question. Moreover, some observers will, and indeed already have [14], argued that altruistic conservation motives are used as ‘greenwashing’ to advance commercial interests in agribusiness and human reproduction. Regardless of the validity of such accusations, de-extinction will need to address this prominent impression.

Here there is an important lesson again in past experiences with cloning endangered animals. Some zoo scientists saw the mass media's positive portrayals of technology as a means to bring new forms of funding into the zoo from wealthy benefactors who are excited about the potential of new technologies [9]. To generate this kind of funding, the animals chosen for these experiments are those that are most likely to receive positive media attention.

This process is also seen in de-extinction, where the charismatic animals used to support ‘cool,’ new de-extinction technologies include the woolly mammoth, the passenger pigeon, and the saber-toothed cat. The concern in the context of cloning endangered animals was that this funding at times drove the science, rather than species preservation itself [9]. The lesson for de-extinction is that financial interest is not the only political economy question that needs to be addressed. Rather, funding itself is constitutive of how experiments are designed. In cloning endangered animals, alternative funding sources have been pursued in part in order to do cloning in ways that have clearer implications for present-day species preservation [9].

## Conclusion

De-extinction illustrates a more general trend toward promissory communication, where scientists promote their work by talking about things that have not happened yet, and may never happen. Discussions detached from what is actually realizable today or in any near future stimulate ‘speculative ethics’ [15],[16]. Proponents and critics alike end up devoting a considerable amount of time and effort to debating the consequences of a science that is yet to be realized. In contrast to speculative ethics, we propose a social science approach based upon the current realities of cloning, genetic engineering, back breeding, and species preservation today. Seemingly mundane questions about matters like husbandry and everyday lab practices are prioritized here, and could be useful to address as de-extinction moves forward. In this context, we argue that social scientists should be included in discussions regarding de-extinction. This would diffuse the spectacle of de-extinction and make it mundane, refocusing attention onto questions about why and how certain species are being resurrected through such programmes and the kinds of lives these animals will be made to live.
